# Effect of soy milk on circulating 17- β estradiol, number of neurons in cerebral cortex and hippocampus and determination of their ratio in neonatal ovariectomized rats

**Published:** 2016-12-15

**Authors:** Behrokh Marzban Abbasabadi, Mina Tadjalli

**Affiliations:** 1 Department of Basic Veterinary Sciences, Faculty of Veterinary Medicine, Amol University of Special Modern Technologies, Amol, Iran; 2 Department of Anatomical Sciences, School of Veterinary Medicine, Shiraz University, Shiraz, Iran

**Keywords:** 17- β estradiol, Hippocampus, Neuron, Rat, Soy milk

## Abstract

This study was conducted to evaluate the effect of soy milk on serum 17- β estradiol level and number of neurons in cerebral cortex and hippocampus as well as determination of the ratio of neurons in cortical and hippocampal regions in neonatal ovariectomized rats. Thirty female rats (one day old) were divided into six groups of five. At day 7, ovariectomy surgery was performed in four groups and two other groups were assumed as sham and control groups. Three groups of ovareictomaized rats were fed with soy milk at the doses of 0.75, 1.50 and 3.00 mL kg^-1 ^per day since they were 14. At day 60, the blood samples were collected to measure the17- β estradiol concentration, and then the brain of rats were prepared for histological studies. The serum 17- β estradiol level significantly increased in ovariectomized rats fed with soy milk compared to ovariectomized rats with no soy milk supplementation. In addition, the results showed that soy milk significantly increased the number of neurons in CA1, CA2 and dentate gyrus regions of hippocampus and granular layer of cerebral cortex in ovariectomized rats, whereas there was no significant change in number of neurons in CA3 zone of hippocampus and molecular, pyramidal and multiform layers of cerebral cortex in ovariectomized rats fed with soy milk. The ratio of cerebral cortex neurons to hippocampal neurons had no significant changes among the experimental groups.

## Introduction

Studies examining dietary intake between various populations have found a vast difference in the amount of isoflavones consumed in Western vs. Asian diets.^1^ These studies have shown that there is an association between the higher consumption of soy products and lower prevalence of hormone-related conditions such as breast cancer and hot flashes in Asian women.^2^^-^^4^ Additionally, prevalence rates for Alzheimer disease are significantly lower in Japan and China compared to countries where a Western-style diet is consumed.^5^

The previous studies revealed that soy contains the largest concentration of isoflavones, a class of phytoestrogens. Phytoestrogens are structurally similar to estradiol and mimic its effects.^6^

The data indicate that consumption of phytoestrogens may help to counteract the drop in estrogen levels at menopause.^6^ By extension, this could be anticipated to protect against cognitive decline.

Soy milk popularity has increased in food market and received a widespread attention because of its numerous health benefits.^7^ Therefore, the aim of the present investigation was to study the effect of soy milk on the serum 17- β estradiol level and number of cerebral cortex and hippocampal neurons in ovariectomized rats.

## Materials and Methods

**Animals and experimental design. **Thirty female rats (one day old) and six mothers were obtained from Shiraz University of Medical Sciences. The rats were maintained in 12 hr light/dark cycles and standard temperature 20 to 24 ˚C and were divided into six groups of five, then, for feeding of neonates, one mother was allocated to each group. Group 1 (control) was intact with no supplementation, group 2 included ovariectomized rats (the ovariectomy operation was performed at day seven) with no supplementation, group 3 as sham underwent laparotomy and abdominal manipulation. The rats in group 4, 5 and 6 were underwent ovariectomy operation (the operation was performed at day seven) and received 1.5% soy milk (Lam Soon, Selangor, Malaysia) of soy protein) at the doses of 0.75, 1.50 and 3.00 mL kg^-1^ per day since day 14, respectively. The soy milk contained 1.50% (1.50 g per 100 mL) of soy protein. After two months (day 60), at the end of the experiment, the rats were deeply anesthetized by diethyl ether and then, blood samples were collected for measuring of serum 17-β concentration. After that, buffer formalin 10% was injected into the hearts before brains dissection and insertion in buffer formalin 10 % for 24 hr.

**Determination of serum 17-β estradiol concentration. **The blood concentration of 17-β estradiol was measured by ELISA (BioSource, Nivelles, Belgium).

**Histological study for counting neurons. **The formaldehyde-fixed samples were dehydrated, cleared and embedded in paraffin. Serial sections of 5 μm thicknesses were cut, mounted and stained with hematoxylin and eosin method and examined by a light microscope that was equipped to a graticule. The following criteria were evaluated in all groups: A) the number of neurons in molecular, granular, pyramidal and multiform layers of cerebral cortex; B) the number of neurons in zones of CA1, CA2, CA3 and dentate gyrus of hippocampus; and C) the ratio of neuron numbers in cerebral cortex to the number of neurons in hippocampus.

**Ovariectomy operation.** Ovariectomy was done on 7^th^ day of birth. The rats were anesthetized by intraperitoneal ketamine (100 mg kg^-1^, Alfasan, Woerden, The Netherlands),^8^ and restrained on their back and the abdominal wall was prepared for an aseptic surgery. The linea alba was incised and both ovaries were exposed, excised and removed. The abdominal wall was closed routinely.

**Statistical analysis. **Data were analyzed by SPSS (version 16; SPSS Inc., Chicago, USA). The data were analyzed by one way analysis of variance and Duncan as post hoc test and the level of significance was considered *p* < 0.05. The Data were presented as mean ± SEM.

## Results

**Fig. 1:**
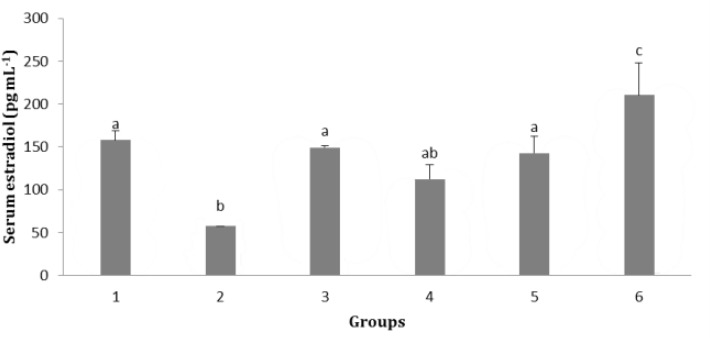
Effect of soy milk on serum 17- β estradiol in ovari-ectomized rats (mean ± SE). **1)** Control; **2)** Ovariectomized rats (no soy milk); **3)** Sham; **4)** Ovariectomized rats treated by soy milk (0.75 mL kg^-1 ^per day); **5)** Ovariectomized rats treated by soy milk (1.50 mL kg^-1^ per day); **6)** Ovariectomized rats treated by soy milk (3.00 mL kg^-1^ per day). The different letters represent significant statistical difference between groups (*p* < 0.05

 **Effect of soy milk supplementation on serum 17- β estradiol concentration. **Our results showed that the serum 17- β estradiol concentration in group 2 was significantly lower than that of control, sham and group 5 and 6 and the highest significant increase was observed in group 6 compared to the others (*p* < 0.05; Fig. 1).

**Fig. 2:**
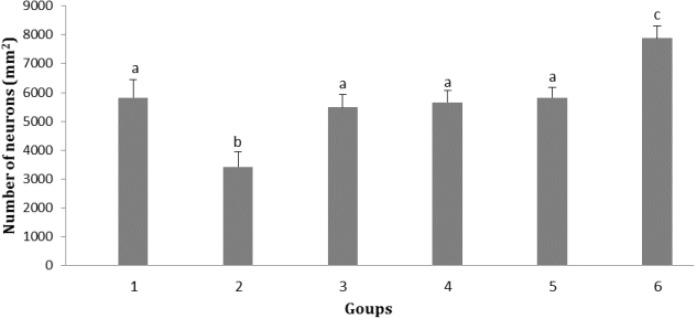
Effect of soy milk on number of neurons in granular layer of cerebral cortex in ovariectomized rats (mean ± SE). **1)** Control; **2)** Ovariectomized rats (no soy milk); **3)** Sham; **4)** Ovariectomized rats treated by soy milk (0.75 mL kg^-1^ per day); **5)** Ovariectomized rats treated by soy milk (1.50 mL kg^-1^ per day); **6)** Ovariectomized rats treated by soy milk (3.00 mL kg^-1^ per day). The different letters represent significant statistical difference between groups (*p* < 0.05

**Effect of soy milk supplementation on number of neurons in cerebral cortex. **Results showed that there was no significant change in number of neurons in molecular, pyramidal and multiform layers of cerebral cortex among different groups, while the number of neurons in granular layer of cerebral cortex in group 2 was significantly lower than that of other groups (*p* < 0.05). A significant  increase in number of neurons in this layer was observed in group 6 which received the highest amount of soy milk (3.00 mL kg^-1^ per day) compared to other groups (*p* < 0.05; Fig. 2).

**Fig. 3:**
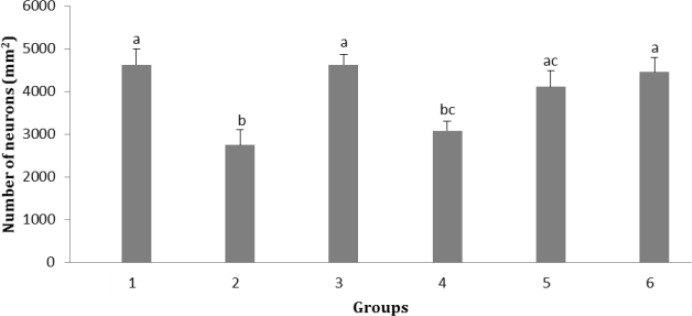
Effect of soy milk on number of neurons in CA1 zone of hippocampus in ovariectomized rats (mean ± SE). **1)** Control; **2)** Ovariectomized rats (no soy milk); **3)** Sham; **4)** Ovariectomized rats treated by soy milk (0.75 mL kg^-1^ per day); **5)** Ovariectomized rats treated by soy milk (1.50 mL kg^-1^ per day); **6)** Ovariectomized rats treated by soy milk (3.00 mL kg^-1^ per day). The different letters represent significant statistical difference between groups (*p* < 0.05

**Effect of soy milk supplementation on number of neurons in different zones of hippocampus.** The number of neurons in CA1 zone of hippocampus in groups 2 and 4 was significantly lower than that of control, sham and group 6 (*p* < 0.05). Also, number of neurons in this zone in group 2 was significantly lower than that of group 5 which fed by 1.50 mL kg^-1 ^per day soy milk (*p *< 0.05; Fig. 3).

**Fig. 4:**
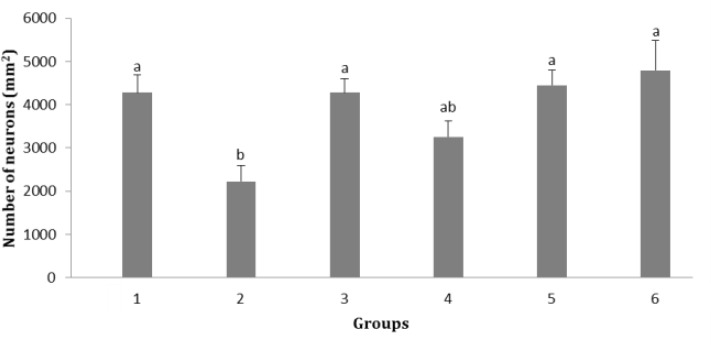
Effect of soy milk on number of neurons in CA2 zone of hippocampus in ovariectomized rats (mean ± standard error). **1)** Control; **2)** Ovariectomized rats (no soy milk); **3)** Sham; **4)** Ovariectomized rats treated by soy milk (0.75 mL kg^-1^ per day); **5)** Ovariectomized rats treated by soy milk (1.50 mL kg^-1^ per day); **6)** Ovariectomized rats treated by soy milk (3.00 mL kg^-1^ per day). The different letters represent significant statistical difference between groups (*p* < 0.05

The number of neurons in CA2 zone of hippocampus in groups 2 was significantly lower than that of control, sham and groups 5 and 6 (*p* < 0.05; Fig. 4). There was no significant change in neuron numbers in group 4 compared to that of other groups. The results showed that there was no significant change in number of neurons in CA3 zone of hippocampus among all groups.

Number of neurons in dentate gyrus zone of hippocampus in groups 2 was significantly lower than that of control and group 5 and 6 (*p* < 0.05). Also, the number of neurons in dentate gyrus zone of hippocampus in group 6 was significantly higher than that of other groups (*p *< 0.05).

**Effect of soy milk supplementation on ratio of number of neurons in cerebral cortex to number of neurons in hippocampus.**The soy milk had no significant effect on the ratio of neuron numbers in cerebral cortex to that of hippocampus.

## Discussion

 In numerous experimental models of neuro-degeneration, the steroid hormone estrogen has shown a powerful neuroprotective effect.^9^^-^^13^ The hormone may directly affect cell survival or prevent cell death by acting on cell death cascades. In addition, estradiol may promote cell survival by the induction of axonal sprouting of injured axons through augmenting regeneration of damaged neural connections and promoting synaptic transmission.^14^ Soy isoflavones, known as phytoestrogens, have estrogenic and anti-estrogenic properties.^15^ They have structural similarities with mammalian estrogens and may interact with pathways of estrogen activity in the body.^16^

Our results showed that serum 17- β estradiol concentration in ovariectomized rats fed with soy milk at the dose of 1.50 mL kg^-1^ per day had no significant changes compared with that of control and sham groups, but the serum 17- β estradiol concentration in ovariectomized rats fed with 3.00 mL kg^-1 ^per day soy milk was significantly higher than that of control and sham groups (*p* < 0.05). It has been reported that diet containing isoflavones leads to reduction of estradiol catabolism by 21.00 to 26.00%. Since estrogen circulates predominately as inactive sulfates which are deconjugated within peripheral tissues, it selectively catabolized and excreted.^16^ As soy isoflavonoids are metabolized in similar enzymatic pathway to estrogen like phytoestrogen, it has been suggested that exposure to certain isoflavones may modulate pathway of estrogen catabolism. Dietary phytoestrogens may modulate the consequences of the postmenopausal estrogen deficiency state.^17^ Our findings indicated that soy milk diet had no significant effect on neuron numbers of molecular, pyramidal and multiform layers of cerebral cortex, although in ovariectomized rats fed with soy milk at the doses of 0.75, 1.50 and 3.00 mL kg^-1 ^per day, the number of neurons was higher than that of ovariectomized rats with no soy milk supplementation. In addition, the number of neurons in granular layer of cerebral cortex was significantly higher in ovariectomized rats received soy milk (3.00 mL kg^ -1 ^per day) than that of other groups. The number of neurons in cerebral cortex was increased as the dose of soy milk was increased. It has been reported that ovariectomized rats given a high dose of soy diet for two weeks proved potentially beneficial increases in growth factor receptor gene expression in several brain areas.^9^ It has been shown that both estradiol and a high soy diet increase brain derived neurotrophic factor mRNA levels in the frontal cortex of retired breeder female rats.^18^

Our findings revealed that soy milk diet significantly increased the number of neurons in CA1, CA2 and dentate gyrus regions of hippocampus. In CA3 zone, although an increase in the number of neurons in groups 4, 5 and 6 was observed but it was insignificant. There is a strong agreement that spatial memory is dependent on the integrity of the hippocampus.^19^^-^^21^ Previous studies have reported that phytoestrogens are neuroprotective.^22^^-^^27^ and increase memory and cognitive function in rats.^28^^,^^29^ It was found that the brains of old rats retain the ability to increase the production of new cells in response to estradiol and soy extract and also the production of new cells in dentate gyrus.^30^ It has been shown that dietary phytoestrogens enhance spatial memory and spine density in the hippocampus and prefrontal cortex of ovariectomized rats.^31^ Further, it has been reported that phytoestrogen genistein pretreatment ameliorates Aβ-induced impairment of short-term spatial memory in rats through an estrogenic pathway and by inducing oxidative stress attenuation.^32^ The present study showed that the ratio of cerebral cortex neurons to hippocampal neurons had no significant changes.

In conclusion, this study showed that the soy milk supplementation especially in high doses can compensate the low serum estradiol level and the decrease of neuron numbers in brain cortex and hippocampus following ovariectomy in neonate rats.
